# A nomogram for predicting probability of low risk of MammaPrint results in women with clinically high-risk breast cancer

**DOI:** 10.1038/s41598-021-02992-8

**Published:** 2021-12-06

**Authors:** Young Joo Lee, Young Sol Hwang, Junetae Kim, Sei-Hyun Ahn, Byung Ho Son, Hee Jeong Kim, Beom Seok Ko, Jisun Kim, Il Yong Chung, Jong Won Lee, Sae Byul Lee

**Affiliations:** 1 Division of Breast Surgery, Department of Surgery, Seoul St Mary’s Hospital, College of Medicine, The Catholic University of Korea, Seoul, Republic of Korea; 2 Division of Breast Surgery, Department of Surgery, Asan Medical Center, University of Ulsan College of Medicine, Seoul, Republic of Korea; 3 Graduate School of Cancer Science and Policy, National Cancer Center, Goyang-si, Republic of Korea; 4 University of Ulsan College of Medicine, Seoul, Republic of Korea

**Keywords:** Cancer, Oncology

## Abstract

We aimed to develop a prediction MammaPrint (MMP) genomic risk assessment nomogram model for hormone-receptor positive (HR+) and human epidermal growth factor receptor-2 negative (HER2–) breast cancer and minimal axillary burden (N0-1) tumors using clinicopathological factors of patients who underwent an MMP test for decision making regarding adjuvant chemotherapy. A total of 409 T1-3 N0-1 M0 HR + and HER2– breast cancer patients whose MMP genomic risk results and clinicopathological factors were available from 2017 to 2020 were analyzed. With randomly selected 306 patients, we developed a nomogram for predicting a low-risk subgroup of MMP results and externally validated with remaining patients (n = 103). Multivariate analysis revealed that the age at diagnosis, progesterone receptor (PR) score, nuclear grade, and Ki-67 were significantly associated with MMP risk results. We developed an MMP low-risk predictive nomogram. With a cut off value at 5% and 95% probability of low-risk MMP, the nomogram accurately predicted the results with 100% positive predictive value (PPV) and negative predictive value respectively. When applied to cut-off value at 35%, the specificity and PPV was 95% and 86% respectively. The area under the receiver operating characteristic curve was 0.82 (95% confidence interval [CI] 0.77 to 0.87). When applied to the validation group, the nomogram was accurate with an area under the curve of 0.77 (95% CI 0.68 to 0.86). Our nomogram, which incorporates four traditional prognostic factors, i.e., age, PR, nuclear grade, and Ki-67, could predict the probability of obtaining a low MMP risk in a cohort of high clinical risk patients. This nomogram can aid the prompt selection of patients who does not need additional MMP testing.

## Introduction

The decision as to whether administer adjuvant chemotherapy for hormone receptor (HR) positive and human epidermal growth factor receptor 2 (HER2)-negative breast cancer is becoming increasingly complex^1–4^. Genomic analysis would be useful to make a decision in this subgroup of breast cancer patients with intermediate risk of prognostic clinical factors. Most of the breast cancer guidelines introduces to consider several genomic assays regarding the use of adjuvant chemotherapy^5–8^. MammaPrint (MMP), a 70-gene-signature‒based test is available for patients with HR + and minimal nodal involvement (N0-1). Women with clinical pathological low risk, additional chemotherapy was not beneficial regardless of genomic assay results. Also benefit of chemotherapy in patients at high clinical risk and low genomic risk was not significant in distant metastasis free survival with 5 years follow up. Identifying these patients may allow reduce unnecessary cytotoxic treatment^9^. Only limited patients with node metastasis were regarded as clinical low risk (well differentiated T1 tumor) according to the MINDACT study design, most of the node positive patients with high genomic risk requires chemotherapy^10^. Identifying these patients is important that the clinicians should convince the patients who wants to avoid unnecessary chemotherapy which are beneficial to them. However, there might be some obstacles in actually performing genomic assays. For example, the cost of genomic assays differ from approximately 2000 US$ to 4000 US$ by products in South Korea and is not covered by national health insurance which can be a significant burden for patients who are not covered by additional private medical insurance. The time from surgery to obtaining the result of assay might also be a concern especially for sending specimens overseas. Ordering MMP usually requires pathology report from the requested healthcare center especially with tumor characteristics. Considering that average time from surgery to pathologic report is about 7 ~ 10 days, the total duration expected to get results is about one month or slightly more. This delay may lead to a negative impact on sufficient decision-making between clinicians and patients. We had created a nomogram for predicting Oncotype DX Recurrence Score 3 years ago^11^, initiated an effort to provide clinicians with an easier decision making process regarding adjuvant chemotherapy. However, considering that most of the patients included in the previous study were node negative, developing a new model with node positive patients is necessary.

The purpose of this study is to develop a nomogram model for MMP genomic risk assessment in patients with hormone receptor positive, HER2 negative, and minimal axillary burden (N0-1) breast cancer by using widely used clinicopathological factors for predicting breast cancer outcomes in a subset of patients subjected to the MMP test to enable prompt screening of patients with extremely high chance of receiving either low or high.genomic risk results.

## Materials and methods

### Patient selection and pathology variable selection

The primary cohort was selected through the evaluation of the initial record of all T1-3N0-1M0 hormone receptor-positive and HER2-negative breast cancer patients whose tumor tissues were analyzed using the MMP test between 2017 and 2020 at Asan Medical Center, Seoul, Republic of Korea. A study data set of 409 cases with available MMP test results was used to build the prediction models. The clinical data of the patients were obtained from the electronic medical records. Clinical information, including patient age, tumor size, lymph node status, pathological stage, histological grade, nuclear grade, lymphovascular invasion (LVI), Ki-67, p53, and molecular subtypes according to the ER, PR, and HER2 status based on immunohistochemistry with or without fluorescence in situ hybridization were obtained. Immunohistochemistry for ER, PR, HER2, and Ki-67 and in situ hybridization for HER2 were performed at the Asan Medical Center pathology laboratories. Nuclear staining for ER and PR was evaluated using the Allred scoring method (0–8). Membrane staining for HER2 was evaluated using the HercepTest (BenchMark XT autostainer using OptiView DAB Detection Kit, Ventana Medical Systems, Tucson, AZ) protocol. Immunohistochemistry for Ki-67 (1:250, MIB-1, Dako, Glostrup, Denmark) was performed using a BenchMark XT autostainer (Ventana Medical Systems) with an i-View detection kit (Ventana Medical Systems).

### Statistical analysis

MMP test results were categorized as low or high genomic risk. Chi-square test and Fisher’s exact test were used to compare the MMP results among clinicopathological characteristics. From primary cohort of 409 patients, we randomly divided the data into two groups at a ratio of 3:1. The larger group was used to develop predictive model and the other used to validate the model externally. Chi-square test and Fisher’s exact test were used to compare the MMP results among clinicopathological characteristics. Initial variable selection was performed on the basis of univariate linear regression in development samples. Four factors, including age at diagnosis (20–100), nuclear grade (range, 1‒3), Allred scores for PR status (range, 0‒8) and Ki-67 labeling index (percentage, 0‒100) were found to contribute significantly by the multivariate logistic regression model. Using this identified factors, we developed a nomogram for predicting probability to achieve genomic low risk. The nomogram was validated both internally and externally with two groups divided. We employed the receiver operating characteristic curve analysis and calculated the area under the curve (AUC). All data analyses were performed using R statistical package ver. 3.2.0 (http://r-project.org). Significance level was set at 0.05 and all p-values were two-sided.

### Ethical statement

The project was reviewed and approved by the Asan Medical Center institutional review board (2020-0037). Due to the retrospective nature of the study, the requirement for informed consent was waived by Asan Medical Center institutional review board. All methods were performed in accordance with the relevant guidelines and regulations.

## Results

### Baseline characteristics

A detailed comparison of the clinical characteristics of all patients (n = 409) based on the MMP results is shown in Table 1. The average age at diagnosis was 53.3 ± 9.3 years in the MMP low-risk group vs. 47.9 ± 9.8 years in the MMP high-risk group (*p* < 0.001) and the average tumor size was 2.2 ± 1.1 cm vs. 2.4 ± 1.0 cm, *p* = 0.018. In the MMP high-risk group, a significantly higher rate of histological and nuclear grade and a higher Ki-67 level (≥ 20%), all *p* < 0.001, were observed. With respect to the receptor status, most of the patients had a strong Allred score (7–8) of ER. In the MMP high-risk group, 6 patients (3.6%) had an intermediate ER score (5‒6) compared with 2 (0.8%), in the low-risk group. None of the patients had a negative (0‒2) or weak positive (3‒4) ER score. MMP low-risk group had a better strong PR status (7‒8) rate (68.8%) compared with the MMP low-risk group (53.3%) and a lower rate of negative or weak PR status, *p* < 0.001. No difference was seen in the surgical methods. More T2 tumors in the high-risk group (46.2% vs. 58.7%, *p* = 0.045) without any significant difference in the pathological N stage and final stage were observed. There was no significant difference in the number of positive nodes, *p* = 0.942; however, MMP high-risk group had a slightly larger size of positive nodes (5.4 ± 3.9 mm vs. 6.3 ± 4.8 mm, *p* = 0.042). More women in the high-risk group were premenopausal (66.9%, *p* < 0.001).

**Table 1:** Baseline characteristics of the patient cohort.

Variables	MMP low-risk	MMP high-risk	*p*-value
	(N = 240)	(N = 169)	
	N (%)	N (%)	
Age at diagnosis (mean ± SD)	53.3 ± 9.3	47.9 ± 9.8	< 0.001
**Histological grade**			< 0.001
Grade I	19 (8.0%)	3 (1.8%)	
Grade II	218 (90.8%)	139 (82.2%)	
Grade III	3 (1.2%)	27 (16.0%)	
**Nuclear grade**			< 0.001
Grade I	2 (0.8%)	0 (0.0%)	
Grade II	235 (97.9%)	140 (82.8%)	
Grade III	3 (1.3%)	29 (17.2%)	
**Estrogen receptor**			< 0.001
Negative	0 (0.0%)	0 (0.0%)	
Weak	0 (0.0%)	0 (0.0%)	
Intermediate	2 (0.8%)	6 (3.6%)	
Strong	238 (99.2%)	163 (96.4%)	
**Progesterone receptor**			< 0.001
Negative	16 (6.6%)	21 (12.4%)	
Weak	12 (5.0%)	25 (14.8%)	
Intermediate	47 (19.6%)	33 (19.5%)	
Strong	165 (68.8%)	90 (53.3%)	
**Lymphovascular invasion**			< 0.001
Negative	147 (61.2%)	67 (40.4%)	
Positive	93 (38.8%)	99 (59.6%)	
**p-53**			< 0.001
0	78 (32.5%)	56 (33.1%)	
1	109 (45.4%)	51 (30.2%)	
2	43 (17.9%)	33 (19.5%)	
3	10 (4.2%)	29 (17.2%)	
**Ki-67 level**			< 0.001
Low Ki-67 < 20%	177 (73.8%)	57 (33.7%)	
High Ki-67 ≥ 20%	63 (26.2%)	112 (66.3%)	
**Breast surgery**			0.675
Total mastectomy	69 (28.8%)	44 (26.3%)	
Breast conservation surgery	171 (71.2%)	123 (73.7%)	
**Axillary operation**			0.056
Axillary dissection	3 (1.3%)	0 (0.0%)	
Sentinel node biopsy	133 (55.6%)	78 (46.7%)	
Axillary dissection after sentinel node biopsy	103 (43.1%)	89 (53.3%)	
**T stage**			0.045
T1	124 (51.7%)	67 (40.1%)	
T2	111 (46.2%)	98 (58.7%)	
T3	5 (2.1%)	2 (1.2%)	
**N stage**			0.416
N0	9 (3.8%)	10 (6.0%)	
N1	231 (96.2%)	157 (94.0%)	
**Stage**			0.449
Stage I	31 (12.9%)	16 (9.6%)	
Stage II	204 (85.0%)	149 (89.2%)	
Stage III	5 (2.1%)	2 (1.2%)	
Tumor size (cm) (mean ± SD)	2.2 ± 1.1	2.4 ± 1.0	0.018
**Number of positive nodes**			0.942
0	9 (3.8%)	8 (4.8%)	
1	150 (62.5%)	105 (62.9%)	
2	66 (27.5%)	45 (26.9%)	
3	15 (6.2%)	9 (5.4%)	
Largest positive node size (mm)	5.4 ± 3.9	6.3 ± 4.8	0.042
**Menopausal status**			< 0.001
Premenopause	112 (46.7%)	113 (66.9%)	
Postmenopause	128 (53.3%)	55 (32.5%)	
Unknown	0 (0.0%)	1 (0.6%)	

MMP, MammaPrint; SD, standard deviation.

### Development of model predicting MMP results

**Figure 1:**
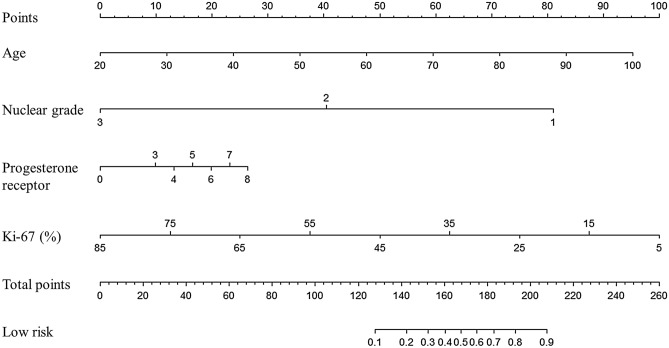
Nomogram to predict a MammaPrint low risk. Age, Progesterone receptor, nuclear grade, and Ki-67 levels were finally selected to develop the model.

**Figure 2:**
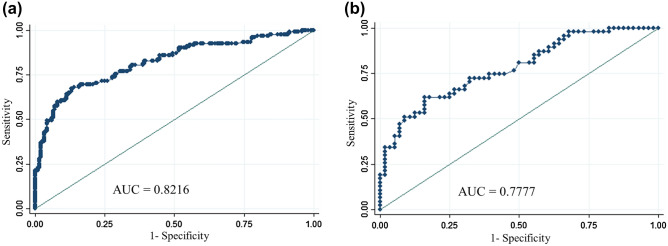
Receiver operating characteristic curve of nomogram. (**a**) Training group of 312 patients. (**b**) Validation group of 97 patients.

We randomized the data into two groups of random sizes of 306 patients at a ratio of 3:1 to develop nomogram. Detailed clinical characteristics of patients included in the development cohort and validation cohort (n = 103) are shown in Table 2. There were no significant differences in the clinical characteristics between these two cohorts. In multivariate analysis, patient age at diagnosis, nuclear grade, PR, and Ki-67 were all found to be independent predictors of MMP genomic low risk. The odds ratio and coefficient associated with four significant factors in the multivariate model are shown in Table 3. A strong PR status was a positive effect, and a higher nuclear grade, younger age, and a higher Ki-67 were negative effects to MMP genomic low risk (Fig. 1). The effect each four factors were converted into points to calculate total points which shows probability of achieving MMP genomic low risk (Fig. 1, Supplement Table S1). The overall predictive accuracy of the nomogram was measured based on the AUC, which was 0.82 (95% confidence interval [CI], 0.77 to 0.87) for the training dataset of 306 patients, and 0.77 (95% CI, 0.68 to 0.86) for the internal validation dataset of 103 patients (Fig. 2).

**Table 2:** Characteristics of validation and training groups.

Variables	Validation set	Training set	*p-*value
	(N = 103)	(N = 306)	
	N (%)	N (%)	
Age at diagnosis (mean ± SD*)	51.2 ± 10.2	51.0 ± 9.8	0.893
**MammaPrint results**			0.496
Low risk	57 (55.3%)	183 (59.8%)	
High risk	46 (44.7%)	123 (40.2%)	
**Histological grade**			0.949
Grade I	6 (5.8%)	16 (5.2%)	
Grade II	90 (87.4%)	267 (87.3%)	
Grade III	7 (6.8%)	23 (7.5%)	
**Nuclear grade**			0.656
Grade I	1 (1.0%)	1 (0.3%)	
Grade II	95 (92.2%)	280 (91.5%)	
Grade III	7 (6.8%)	25 (8.2%)	
**Estrogen receptor**			0.685
Negative	0 (0.0%)	0 (0.0%)	
Weak	0 (0.0%)	0 (0.0%)	
Intermediate	1 (1.0%)	7 (2.3%)	
Strong	102 (99.0%)	299 (97.7%)	
**Progesterone receptor**			0.132
Negative	15 (14.6%)	22 (7.2%)	
Weak	7 (6.8%)	30 (9.8%)	
Intermediate	20 (19.4%)	60 (19.6%)	
Strong	61 (59.2%)	194 (63.4%)	
**Lymphovascular invasion**			0.772
Negative	52 (51.0%)	162 (53.3%)	
Positive	50 (49.0%)	142 (46.7%)	
**p-53**			0.277
0	40 (38.8%)	94 (30.7%)	
1	40 (38.8%)	120 (39.2%)	
2	17 (16.5%)	59 (19.3%)	
3	6 (5.8%)	33 (10.8%)	
**Ki-67 level**			0.718
Low Ki-67 < 20%	61 (59.2%)	173 (56.5%)	
High Ki-67 ≥ 20%	42 (40.8%)	133 (43.5%)	
**Breast surgery**			1.000
Total mastectomy	29 (28.2%)	84 (27.6%)	
Breast conservation surgery	74 (71.8%)	220 (72.4%)	
**Axillary operation**			0.947
Axillary dissection	1 (1.0%)	2 (0.7%)	
Sentinel node biopsy	53 (51.5%)	158 (52.1%)	
Axillary dissection after sentinel node Biopsy	49 (47.6%)	143 (47.2%)	
**T stage**			0.736
T1	47 (45.6%)	144 (47.4%)	
T2	55 (53.4%)	154 (50.7%)	
T3	1 (1.0%)	6 (2.0%)	
**N stage**			0.480
N0	3 (2.9%)	16 (5.3%)	
N1	100 (97.1%)	288 (94.7%)	
**Stage**			0.795
Stage I	12 (11.7%)	35 (11.5%)	
Stage II	90 (87.4%)	263 (86.5%)	
Stage III	1 (1.0%)	6 (2.0%)	
Tumor size (cm) (mean ± SD)	2.2 ± 1.1	2.3 ± 1.1	0.734
**Number of positive node**			0.262
0	1 (1.0%)	16 (5.3%)	
1	64 (62.1%)	191 (62.8%)	
2	31 (30.1%)	80 (26.3%)	
3	7 (6.8%)	17 (5.6%)	
Largest positive node size (mm)	6.5 ± 4.9	5.5 ± 4.1	0.064
**Menopausal status**			0.776
Premenopause	55 (53.4%)	170 (55.6%)	
Postmenopause	48 (46.6%)	135 (44.1%)	
Unknown	0 (0.0%)	1 (0.3%)	

SD, standard deviation.

**Table 3:** Multivariate logistic regression model.

Variables	Multivariate model				
	*β*-coefficient	Standard error	Z score	*p-*value	95% confidence interval
Age	0.653554	0.016	3.92	0.000	0.032–0.098
Progesterone receptor status	0.1812972	0.780	− 5.61	0.005	0.054–0.307
Nuclear grade	− 2.223743	0.064	− 2.85	0.004	− 3.754–0.693
Ki-67	− 0.0686273	0.012	1.06	0.000	− 1.687–0.044

### Sensitivity, specificity and positive and negative predictive values

Table 4 shows the sensitivity, specificity, and positive and negative predictive values according to various cutoff values. Each probability shown in the table represents the cutoff value of different probabilities of obtaining a MMP genomic low result; for example, when we apply a probability of 90% as a cutoff for binary risk results, it means that the nomogram results higher than 90% will have a high chance of being associated with actual MMP low risk. In the opposite way, nomogram results of less than probability of 10% indicates a very low chance to get actual low risk. Based on our results, use of a low cut off value of 5% strongly predicted the actual MMP high risk, with 100% positive predictive value (PPV) and specificity.

**Table 4:** Sensitivity, specificity, positive predictive, and negative predictive values according to various cutoff values.

Cutoff value of calculated probability achieving MMP low risk (%)	Risk assessment by nomogram	Number of total patients (N = 306)	Number of patients with High risk MMP (N = 123)	Number of patients with Low risk of MMP (N = 183)	Sensitivity (%)	Specificity (%)	PPV (%)	NPV (%)
5	High risk	16	16	0	13	100	100	63
	Low risk	290	107	183				
10	High risk	31	28	3	22	98	90	65
	Low risk	275	95	180				
30	High risk	53	47	6	38	96	88	69
	Low risk	253	76	177				
35	High risk	61	53	8	43	95	86	71
	Low risk	245	70	175				
50	High risk	94	74	20	60	89	78	76
	Low risk	212	49	163				
70	High risk	154	94	60	76	67	61	80
	Low risk	152	29	123				
88	High risk	259	116	143	94	21	44	85
	Low risk	47	7	40				
90	High risk	275	119	156	96	14	43	87
	Low risk	31	4	27				
95	High risk	302	123	179	100	2	40	100
	Low risk	4	0	4				

MMP, Mammprint test; PPV, positive predictive value; NPV, negative predictive value.

### User-friendly calculator

**Figure 3:**
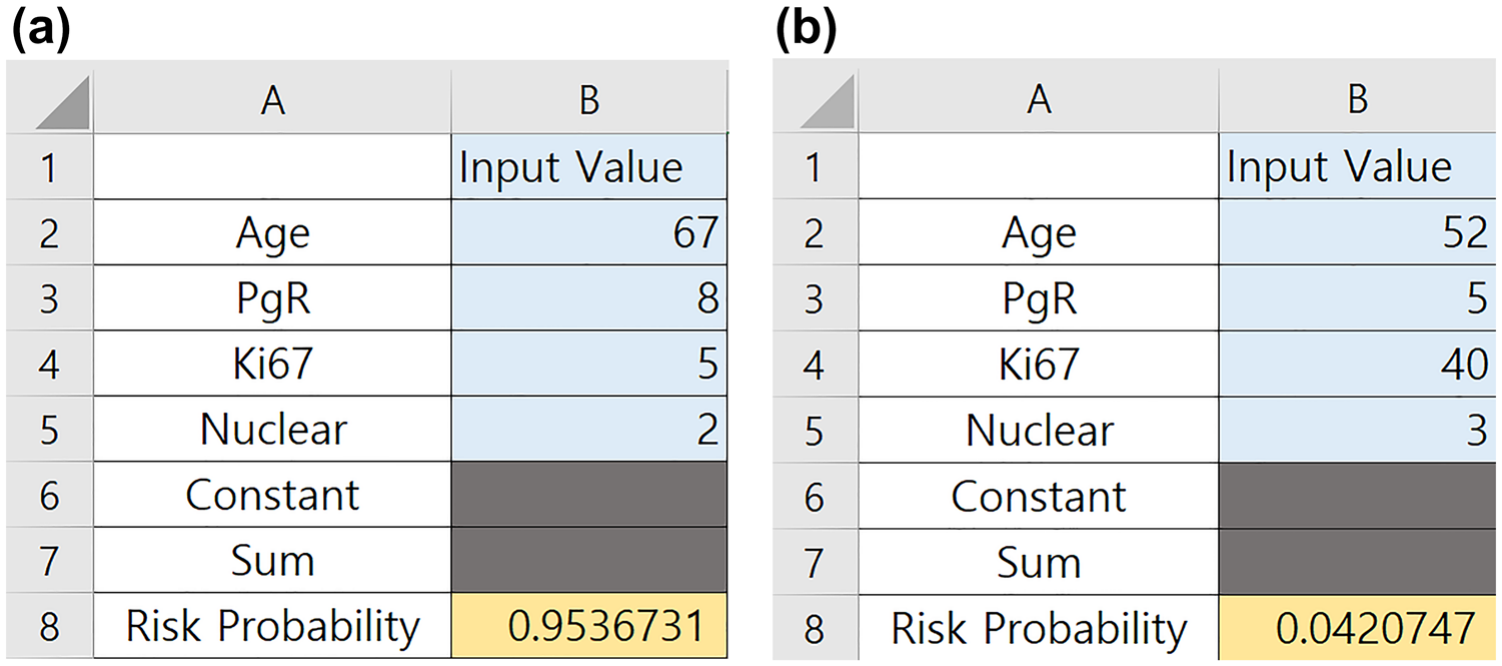
Automatic calculator using Microsoft Excel worksheets. (**a**) 95% probability of low Mammaprint risk with age 67, strong PR (8), low Ki67 (5%) and tumor grade 2. (**b**) 4% probability of low Mammaprint risk with age 52, intermediate PR (5), high Ki67 (40%) and high tumor grade.

The results obtained from our analysis were used to develop a user-friendly calculator using Microsoft Excel worksheets (Supplement Excel file). The interface allows the user to input the values of age at diagnosis, PR Allred score (0‒8), Ki-67 level as a percentage, and nuclear grade (1‒3). The standard output includes an estimate of the probability of MMP genomic low risk when actually tested. For example, given the same condition with node positive (1–3) and strong ER status, a woman with age 67, strong PR (8), low Ki67 (5%) and tumor grade 2 will have more than 95% chance to receive actual MMP genomic low risk (Fig. 3a.). Whereas a woman with age 52, intermediate PR (5), high Ki67 (40%) and high tumor grade will have less than 5% to get actual MMP genomic low risk (Fig. 3b). Our algorithm seems to help clinicians in identifying patients with a higher chance of getting a low risk or high risk MMP test and for whom gene testing accessibility is low.

## Discussion

MMP is the one of the most widely used genomic assays for breast cancer testing in the world, especially for patients with one to three positive nodes. The recent National Comprehensive Cancer Network (NCCN) guideline recommends clinicians to consider MMP testing for decision making regarding adjuvant systemic chemotherapy with evidence of category 1, for patients with ER/PR positive, HER2 negative, and with negative or positive (1‒3) node metastasis breast cancer^12^. Although according to interim analysis of RxPONDER trial, the guideline recommends Oncotype DX for same subset of patients, however the interpretation of the results in premenopausal patients is complex^13^. The use of genomic signatures is recommended for this subset of patients by national and international clinical guidelines, i.e., St. Gallen Consensus Conference, European Society for Medical Oncology (ESMO), and American Society of Clinical Oncology^8,14,15^. However, the economic burden on patients makes clinicians hesitate to recommend genomic testing. Although reports on the cost effectiveness of MMP show that MMP safely guided chemotherapy de-escalation in clinical high-risk patients with HR + /HER2- tumors compared (to clinical assessment alone)^16^, the cost of MMP in South Korea (approximately $3,200) can still be a burden for patients. As this test originally designed in foreign countries, the South Korea National Health Insurance does not cover the test, only a few private health insurance companies cover MMP. Therefore, MMP imposes an economic burden in South Korea, regardless of whether patients have private insurance or not. This study was performed to evaluate MMP risk assessment, which was based on routine standard patient and tumor characteristics. Similar prediction models have been developed using clinicopathological data^17^ or radiological phenotype results^18^. However, the best way to link these results with clinical practice has not yet been identified. We found that using this prediction tool with simple four combined clinicopathological factors can promptly screen a subset of patients who actually do not necessarily require costly genomic tests. Finding these groups of patients with undoubtedly high probability of obtaining a low genomic risk (or a high risk) might be useful in situations where genomic testing is unfavorable to perform.

Four clinicopathological variables were used in our model, age, nuclear grade, PR, and Ki-67. The values for the latter three variables can be easily determined through examination in any pathological laboratory in health care centers and were used to predict prognosis before the gene testing era. Here, the Allred score of ER was not included in the final model because most of the patients had high (7‒8) ER scores with variation in the PR status only. This implies that clinicians have tendency to perform fewer genomic tests to decide on adjuvant chemotherapy administration for patients with weak to intermediate ER scores (3‒6)^19,20^. A higher ER status is related to a higher endocrine response and a lower chemotherapy response^21^. In contrast, a low ER status is known to be associated with a low chemotherapy response, which is similar to negative ER tumors, compared with strong ER-positive tumors in neoadjuvant settings^22^. PR status can also be used to predict the endocrine and chemotherapy responses. By analyzing 77 invasive breast cancers and their PR status and 21-gene testing recurrence score results, a strong negative correlation between both factors was revealed^20^.

Another important clinical factor according to our model was the age at diagnosis. A negative association was observed between younger patients and a high nomogram score. When calculated with our nomogram alone, probability of low genomic risk for younger patients (< 50) did not reach 90% with strong PR (8), low Ki-67 (5%) and tumor grade 2. The only chance that younger patients (< 50) reach the probability of more than 95% of low genomic risk probability was with low tumor grade and relatively high PR status and low Ki-67. The chemotherapy benefit for invasive disease-free survival varied when the recurrence score was combined with age (*p* = 0.004), with some chemotherapy benefits found in women < 50 years with a recurrence score of 16–25^5^. According to a recent update on the long-term results of MINDACT trial (EORTC 10,041/BIG3-04) presented at the American society of clinical oncology annual meeting in 2020, there is an absolute 5% ± 2.8% distant metastasis free survival gain with adjuvant chemotherapy in premenopausal women with a discordant clinical and genomic risk (clinical high risk/genomic low risk)^9^. These results lead us to be more careful with omitting chemotherapy in premenopausal patients. Applying our nomogram to premenopausal women, it is appropriate to set the highest cut-off value more than 95% strictly to avoid misclassifications to low genomic risk.

In our previous study on a prediction model for Oncotype DX recurrence score^12^, Ki-67 was most strongly related to the Recurrence Score. The role of Ki-67 as an indicator of poor prognosis in the Oncotype Dx gene assay is well-known^23–25^. Similarly, a current study also revealed that a higher Ki-67 level was closely associated with MMP high risk. Ki-67 itself can be a strong prognostic index; however, recent analyses on intermediate Ki-67 and MMP results showed that for the patients with a low Ki-67 (< 15%) or a high Ki-67 (> 30%), the risk results of the MMP test mostly agreed with the Ki-67 level, while for the patients with an intermediate Ki-67 value (15‒30%), they were discordant with the MMP risk result^26,27^. It is expected that with a definitely high Ki-67, clinicians would be reluctant to forego adjuvant chemotherapy or make a decision after MMP testing. When we looked up our data closely, in 175 Ki-67 high (≥ 20%) patients, 135 patients (77.1%) had an intermediate Ki-67 level (20‒40) and only 40 patients (22.8%) had a Ki-67 level > 40. Our model seems appropriate to represent this in-between prognostic group. Grade has also been long regarded as a prognostic indicator of breast cancer outcome^28^ by Nottingham Prognostic Index, whose association with genomic assays is proven^29^.

In the study population, the majority of the patients were (94.8%) node positive, with up to three lymph nodes. Node positivity is mostly consistent with high clinical risk according to MINDACT trial study design. The MINDACT results implicate that performing genomic test in clinical low risk group has no significant advantage over clinical high risk group. This study provides probability of MMP results especially within clinical high risk group with easily available four clinical factors. Selecting a group of patients with high chance of having no advantage of performing genomic test is feasible with this nomogram. We also offer a user-friendly interfaced calculator with simple four robust factors which can be widely used by clinicians. This study has also some limitations. Due to its retrospective nature, our study might have selection bias with respect to the nature of the primary population, i.e., patients with a low MMP risk. However, this is also a strength, as this population reflects a subset about which clinicians ponder for performing MMP testing. Also, there is a concern that reproducibility of Ki-67 level due to variability of the assay which has not been validated by the St. Gallen guidelines. Because a prediction nomogram will never produce the same result as a genomic test, we suggest that the purpose of using this nomogram should be to decide on whether to perform genomic test for intermediate risk patients who cannot afford the associated medical expense. Further, the study endpoint was set based on the result of MMP, not the subsequent outcome of the patient. we recommend that clinicians interpret and apply the nomogram results carefully after sufficient agreement with patients.

In conclusion, our nomogram, which predicts probability of MMP low genomic risk result, will be a useful tool to help immediate selection of patients with HR + /HER2- and node-positive tumors who have so low (or high) probability to test negative, that testing could not be worth. Combined with various cut-off value given and choice by users, this nomogram can be a useful substitute for MMP testing in cases where genomic testing can be costly or when testing itself is not available.

## Supplementary Information

Supplementary Information 1.

Supplementary Information 2.

## Data Availability

Data archiving is not mandated but data will be made available on reasonable request.
