# Supplementary material for: A nomogram for predicting probability of low risk of MammaPrint results in women with clinically high-risk breast cancer
Source: Sci Rep. 2021 Dec 6;11:23509. doi: 10.1038/s41598-021-02992-8 (PMC8648770; doi:10.1038/s41598-021-02992-8)
Supplement: Supplementary file 2 — Supplementary Information 2. [file 41598_2021_2992_MOESM2_ESM.docx]

Supplement

Supplement Table 1. Nomogram points corresponding each factor and total points to risk calculated probability of MMP genomic low risk.

| Age | Points | nuclear | Points | PgR | Points | ki67 | Points | Total Points | Low Risk Probability |
| --- | --- | --- | --- | --- | --- | --- | --- | --- | --- |
| 25 | 0 | 1 | 81 | 0 | 0 | 5 | 100 | 108 | 0.05 |
| 30 | 6 | 2 | 41 | 3 | 10 | 15 | 87 | 122 | 0.1 |
| 35 | 12 | 3 | 0 | 4 | 13 | 25 | 75 | 130 | 0.15 |
| 40 | 18 |  |  | 5 | 17 | 35 | 62 | 137 | 0.2 |
| 45 | 24 |  |  | 6 | 20 | 45 | 50 | 142 | 0.25 |
| 50 | 30 |  |  | 7 | 23 | 55 | 37 | 146 | 0.3 |
| 55 | 36 |  |  | 8 | 26 | 65 | 25 | 151 | 0.35 |
| 60 | 42 |  |  |  |  | 75 | 12 | 154 | 0.4 |
| 65 | 48 |  |  |  |  | 85 | 0 | 158 | 0.45 |
| 70 | 54 |  |  |  |  |  |  | 162 | 0.5 |
| 75 | 60 |  |  |  |  |  |  | 165 | 0.55 |
| 80 | 65 |  |  |  |  |  |  | 169 | 0.6 |
| 85 | 71 |  |  |  |  |  |  | 173 | 0.65 |
| 90 | 77 |  |  |  |  |  |  | 177 | 0.7 |
| 95 | 83 |  |  |  |  |  |  | 182 | 0.75 |
| 100 | 89 |  |  |  |  |  |  | 187 | 0.8 |
|  |  |  |  |  |  |  |  | 193 | 0.85 |
|  |  |  |  |  |  |  |  | 202 | 0.9 |
|  |  |  |  |  |  |  |  | 215 | 0.95 |
